# Large-scale use of mosquito larval source management for malaria control in Africa: a cost analysis

**DOI:** 10.1186/1475-2875-10-338

**Published:** 2011-11-08

**Authors:** Eve Worrall, Ulrike Fillinger

**Affiliations:** 1 Liverpool School of Tropical Medicine, Pembroke Place, Liverpool L3 5QA, UK; 2 Department of Disease Control, London School of Hygiene and Tropical Medicine, Keppel Street, London WC1E 7HT, UK; 3 International Centre of Insect Physiology and Ecology, Thomas Odhiambo Campus, Mbita 40305, Kenya

**Keywords:** Malaria, cost analyses, vector control, larval control, source management, *Bacillus thuringiensis israelensis*, *Anopheles gambiae*

## Abstract

**Background:**

At present, large-scale use of two malaria vector control methods, long-lasting insecticidal nets (LLINs) and indoor residual spraying (IRS) is being scaled up in Africa with substantial funding from donors. A third vector control method, larval source management (LSM), has been historically very successful and is today widely used for mosquito control globally, except in Africa. With increasing risk of insecticide resistance and a shift to more exophilic vectors, LSM is now under re-evaluation for use against afro-tropical vector species. Here the costs of this intervention were evaluated.

**Methods:**

The 'ingredients approach' was used to estimate the economic and financial costs per person protected per year (pppy) for large-scale LSM using microbial larvicides in three ecologically diverse settings: (1) the coastal metropolitan area of Dar es Salaam in Tanzania, (2) a highly populated Kenyan highland area (Vihiga District), and (3) a lakeside setting in rural western Kenya (Mbita Division). Two scenarios were examined to investigate the cost implications of using alternative product formulations. Sensitivity analyses on product prices were carried out.

**Results:**

The results show that for programmes using the same granular formulation larviciding costs the least pppy in Dar es Salaam (US$0.94), approximately 60% more in Vihiga District (US$1.50) and the most in Mbita Division (US$2.50). However, these costs are reduced substantially if an alternative water-dispensable formulation is used; in Vihiga, this would reduce costs to US$0.79 and, in Mbita Division, to US$1.94. Larvicide and staff salary costs each accounted for approximately a third of the total economic costs per year. The cost pppy depends mainly on: (1) the type of formulation required for treating different aquatic habitats, (2) the human population density relative to the density of aquatic habitats and (3) the potential to target the intervention in space and/or time.

**Conclusion:**

Costs for LSM compare favourably with costs for IRS and LLINs, especially in areas with moderate and focal malaria transmission where mosquito larval habitats are accessible and well defined. LSM presents an attractive tool to be integrated in ongoing malaria control effort in such settings. Further data on the epidemiological health impact of larviciding is required to establish cost effectiveness.

## Background

Malaria research and control in Africa is seeing unprecedented funding support to scale up much needed interventions. The level of funding has increased six-fold from 2003 to 2009 [1]. Key donor sources are the President's Malaria Initiative (PMI), Global Fund to Fight AIDS, Tuberculosis and Malaria (GFATM) and the Department for International Development (DfID) [2]. Funds are used to support diagnoses and treatment through artemisinin combination therapy (ACT), rapid diagnostic tests (RDT) and intermitted preventive treatment in pregnant women and infants (IPTp/i). Support for vector control is mostly spent on long-lasting insecticidal nets **(**LLINs) and, more recently, through PMI on indoor residual spaying (IRS) [3].

Vector control programmes are being encouraged to develop Integrated Vector Management (IVM) strategies for the control of malaria and other vector borne diseases [4]. In IVM, multiple tools are recommended to increase effectiveness and reduce our dependency on insecticides. Larval source management (LSM) might have the capacity to supplement the prioritized vector control measures since it will attack not only the indoor mosquito populations but also those vectors that remain less affected by LLINs and IRS like the outdoor biting and/or resting *Anopheles arabiensis *or secondary malaria vectors, which are less anthopophilic and sustain low malaria transmission after high LLIN/IRS coverage. Moreover, the wide diversity in the mode of actions of larvicides in combination with environmental modifications and manipulations could be an opportunity to maintain the longevity of widely used active ingredients and offers a means to reduce the overall dependence on insecticides.

Despite its enormous historical successes mosquito larval source management (LSM) remains a largely forgotten and often dismissed intervention for malaria control in Africa [5-15]. One of the reasons LSM is not considered for malaria control is that it is perceived as '*beyond the reach of most resource-deprived communities in sub-Saharan Africa*' creating the impression that LSM is far more expensive than other malaria control interventions [16]. However, with increasing risk of insecticide resistance and a shift to more exophilic vectors in response to insecticides indoors LSM is now under re-evaluation for use in Africa[14,17-27].

Recent studies in rural areas of western Kenya have demonstrated that larval control can reduce the abundance of malaria mosquito larvae and adult females by > 90% [27,28]. Furthermore, vector control with microbial larvicides and LLINs combined, resulted in a two-fold reduction in new malaria infections compared with LLINs alone indicating that the addition of anti-larval measures to LLIN programmes provides substantial additional protection against malaria parasites [28]. Similar results have been shown in the city of Dar es Salaam, Tanzania, where LSM is implemented operationally through the Urban Malaria Control Programme [29]. Successes have also been achieved in Eritrea where LSM is included in an integrated vector management programme that has lead to a decrease in malaria of > 50% [30]. These successes have paved the way for LSM to be included in the Global Malaria Action Plan of the Roll Back Malaria Partnership [31].

This paper aims to complement these efforts by costing the implementation of large-scale LSM using microbial larvicides in three different settings in East Africa. These analyses aim to quantify the resource implications of delivering large-scale LSM in terms of economic costs per person protected per year (pppy) and total economic programme costs. Estimates can be considered alongside similar analyses that have been prepared for large-scale use of LLINs and IRS [32]. This paper also presents programme designs for different eco-epidemiological settings, including staff requirements, management system and responsibilities to provide assistance for planning similar programmes.

## Methods

### Eco-epidemiological settings and LSM programme design

The cost analysis presented here was carried out in 2007. Three settings were included representing different ecologies where LSM programmes had been implemented previously and shown to reduce malaria transmission by 70-90% [26-29]. Specifically, costs were estimated for: (1) a LSM programme in 15 city wards of urban Dar es Salaam, Tanzania, (2) a district wide LSM programme in Vihiga District (in 2009 divided in Vihiga, Emuhaya, Hamisi and Sabatia District), a highly populated area in the western Kenyan highlands, and (3) a LSM programme along the shores of Lake Victoria covering Mbita Division in Suba District (in 2009 divided in Suba and Mbita District, Mbita Division is since located in Mbita District), western Kenya (Table 1).

**Table 1:** Target location summary

Study location			Defined Target Areas for Costing LSM programs				Targeting Strategy
Country	City/ District	Description	Administrative area covered^1^	Total Population^2^	Area in km^2^	Population density/km^2^	
Tanzania	Dar es Salaam	Urban	15 city wards	592, 338	58	10, 289	None
Kenya	Vihiga District	Rural highlands	Vihiga District (total 6 divisions)	609, 324	563	1, 082	Spatial and Temporal
Kenya	Suba District	Rural lakeside	Mbita Division	55, 558	211	263	Spatial

1 Costing in Dar es Salaam is based on ongoing operational program, others are fictive programs based on previous research projects.

2 Vihiga and Mbita population projection based on 1999 census. Kenya 1999 population and housing census. Published 2002 by Central Bureau of Statistics, Ministry of Finance and Planning in Nairobi, Kenya. Dar es Salaam population based on sum of population data for selected wards 2002 Population and Housing Census, National Bureau of Statistics, Tanzania

All settings experience two rainy seasons each year: the longer season with peak rainfall from approximately March to June, and the shorter season between October and December. For costing, a LSM programme was designed, but not actually implemented, for the three defined intervention areas. Programme design decisions and estimates of the quantity of key resources required were informed by the existing operational programme in Dar es Salaam, Tanzania and small-scale research projects which have been implemented in these sites previously [26-28].

#### Urban Dar es Salaam

Dar es Salaam is the largest city in Tanzania; with approximately 2.9 million inhabitants. It has distinctive characteristics of urban malaria ecology and epidemiology. Malaria transmission is seasonal and focal with a moderate average parasite prevalence rate in all-age groups < 10% [29]. Interestingly, malaria vectors in the city appear to have adapted to high coverage with bed nets and improved housing by predominantly feeding outdoors [33]. Thus, insecticide-treated nets confer slightly less protection than in rural areas so additional measures directed at aquatic stages of vector mosquitoes may have a useful role in this and similar urban settings [33]

At the time of this analysis, the Urban Malaria Control Programme (UMCP) was operating at different stages of implementation in 15 city wards of Dar es Salaam (five wards per municipality), an area inhabited by more than 592, 000 people and covering 58 km^2 ^(Table 1). LSM was operational in three of the 15 wards, in the remaining nine wards mapping and mosquito surveillance had been carried out in preparation for the intervention [26,29,34,35]. Programme costs (excluding academic research costs) from operational wards were used to estimate the costs for operational LSM in all 15 wards.

#### Western Kenyan Highlands (rural, high population density site)

Recently there has been a marked increase in malaria in the African highlands, largely due to the rise of drug-resistant strains of *Plasmodium falciparum *parasites [36-38]. The ecology of the western highlands of Kenya supports stable transmission and increasing population pressure has led to agricultural changes creating ideal conditions for vector proliferation [19,39].

Here costs were estimated for a LSM programme for the entire Vihiga District, one of the most densely populated districts in Kenya. This District was divided into six divisions, 26 locations, and 110 sub-locations. The district had an area of 563 km^2 ^and an estimated population of 609, 324 (Table 1, [40]) and its elevation is 1450-1580 m altitude. The cost estimations are based on a programme targeted in time with four months larvicide application during the main transmission season [16,28].

Malaria transmission in the district is seasonal and prone to climate related epidemics. A parasite prevalence survey along a transect found that the proportion of children infected with malaria parasites was 68% in the valley bottom (1450 m), 40% percent at mid-elevations (1500 m) and 27% at the hilltops/summit (1580 m) [41]. Crucially however, 98% of *Anopheles gambiae s.s*. and *Anopheles funestus *were collected in the valley bottoms [41]. This raises the probability that a larval control intervention targeted spatially at the valley bottoms will also prevent the vast majority, if not all, transmission in populations residing further up the valley sides.

#### Rural lakeside area (low population density site)

Mbita Division is situated along the shores of Lake Victoria in western Kenya. It is largely rural and sparsely populated, except for Mbita town. Mbita is one of five administrative divisions of Suba District. It covers approximately 20% (211 km^2^) of the surface area of the District and is home to around 55, 558 people (Table 1). Malaria transmission is moderate but perennial with malaria parasite rates ranging between 20-50% (N. Minakawa, pers. communication). Primary malaria vectors are *An. gambiae s.s*., *An. arabiensis *and *An. funestus*. Their larval habitats are well-defined and are primarily found in close vicinity to the lake shore and close to human habitations [14,42]. A longitudinal study over five years in Mbita showed that weekly LSM throughout this period reduced malaria transmission by > 90% and gave the first indication that such impact might be achieved with moderate costs [27]. Due to the heterogeneous ecology of the different divisions within Suba District and the lack of sufficient baseline knowledge of these, the costing was not extrapolated to the entire district but restricted to Mbita Division. To protect its population, analysis of the topography of the Division revealed that approximately two-thirds of the surface area (140 km^2^) would need to be included for LSM. Population density in the remaining one third is extremely low and at higher altitude, where aquatic habitats are rare and malaria transmission intensity very low. Hence spatial targeting was also assumed to be a viable strategy in this area.

### Costing methodology

The ingredients approach was utilized by first identifying the activities to be costed, and then quantifying the financial and economic cost of carrying out these activities [43]. The economic cost analysis captured all resources used, including donations and volunteers, as well as the opportunity cost of existing inputs (e.g. staff time, equipment, and buildings which are already employed/in existence) and excluded taxes and other transfer payments where no resources are used. The cost of capital items was spread over their useful life at a discounted rate. Economists use discounting to make 'fair' comparisons of programmes whose costs and outcomes occur at different times. For example it allows considering the opportunity cost of tying up resources in capital items and the preference for receiving goods or services sooner rather than later. The economic cost analysis is presented from the provider (or supply) perspective so that the Dar es Salaam financial costing takes perspective of the Government of Tanzania and Vihiga and Mbita financial costing takes perspective of the Government of Kenya. Opportunity costs incurred by the community are not captured; however, these costs would be minimal in a LSM programme operating as described in this paper since all community staff involved in the programme are paid a wage for their time, other involvement would only be allowing access to their property.

### Resource requirements

**Figure 1:**
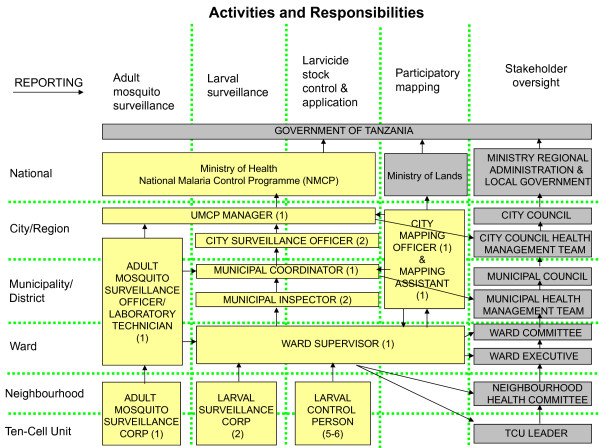
**Model programme structure for an urban LSM programme in Dar es Salaam, Tanzania (modified after **[26]).

**Figure 2:**
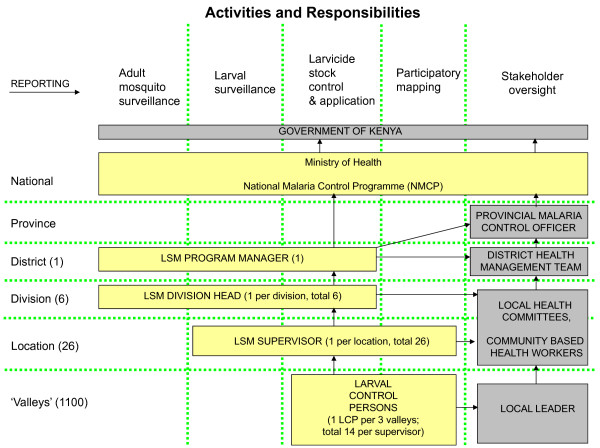
**Model programme structure for a LSM programme in the highland valleys of Vihiga District, western Kenya**.

**Figure 3:**
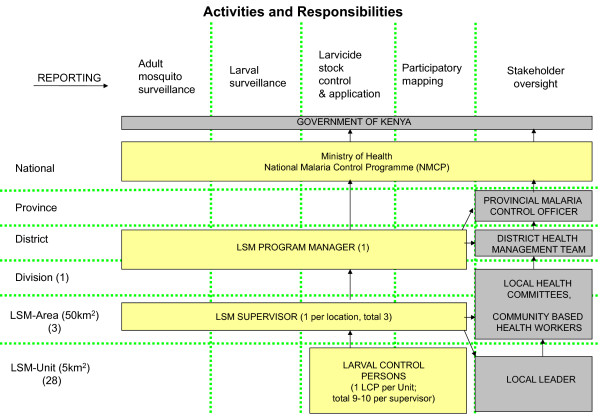
**Model programme structure for LSM along the shores of Lake Victoria in Mbita Division, Suba District, western Kenya**.

Resource requirements were estimated by experts from industry, academia and malaria control programmes in Kenya, and Tanzania working together to describe a larval control programme that would be appropriate for the three settings. The experts involved had significant experience designing and managing larval control programmes. The resource requirements were captured and quantified in a spreadsheet model. The LSM programmes designed (see Table 1, Figures 1, 2 and 3) included a preparatory phase (six months) and an intervention phase (one year). Estimates of the quantity of key resources (e.g. larval control persons (LCPs), supervisors, equipment and larvicide) required were informed by small-scale research projects in the planned intervention areas and the UMCP in Dar es Salaam [26-28]. It is assumed that larvicide manufacturer inputs (technical advice) are provided as part of their user service and, therefore, are implicitly captured in the larvicide product price rather than in staffing. Academic technical expertise was included in the costing for all three programme settings but costs of academic research were excluded.

### Cost estimation

Cost estimates for each LSM component (the 'ingredients') were obtained from a variety of sources, including quotes and invoices for recently purchased equipment. Staff time was valued at full salary costs (mid-range), including allowances according to government pay scales for suitably qualified staff. The opportunity costs of existing Ministry of Health (MoH) employee involvement was estimated based on existing salaries, including allowances. The cost of office and storage space was proxied, based on local rents. The cost of larvicide was based on the midpoint of published ex works 2005 U.S. dollar prices for the formulations (Additional File 1, Table S1: Larvicide Product Prices) [27]. Prices were adjusted to 2006 prices using the U.S. Government estimate of producer price inflation (Additional File 1, Table S2 US Producer price index industry data "all other basic organic manufacturing" sector: price inflator and deflators). Costs were measured in the currency in which they would be paid (specifically, United Kingdom pound [£], United States dollar [US$], Kenya shilling [KES], Tanzania shilling [TZS]), and converted into 2006 U.S. dollars using average exchange rates for 2006 (Additional File 1, Table S3: Exchange Rates). Capital costs were annualized using a discount rate of 3% (Additional File 1, Table S4: Annualization Factors and Discount Rates) and the estimated useful life for specific items (summarized in capital-good unit cost tables in relevant appendices) [44]. Costs incurred during the preparatory phase of each programme were calculated and annualized over their useful life which was assumed to be eight years in line with other studies using a 3% discount rate [44,45]. These were added to the cost of the intervention phase to calculate a typical year's costs.

All costing methodologies were consistently applied across each of the three programme settings. Care was taken to ensure that other assumptions made in the costings were consistent except where programme specific conditions (e.g. vector ecology) necessitated different assumptions.

### Larval control product and formulations

The programmes were costed assuming the use of microbial larvicides for vector control. Commercially available products from Valent BioSciences Corp., Illinois, USA, were used as the basis for costs in this report: VectoBac^® ^(active ingredient: *Bacillus thuringiensis israelensis *(*Bti*)) [46]. The product is available as a water dispersible granule (WG) formulation for liquid application with knapsack sprayers and custom granule (CG) formulations for hand application. These two alternate formulations are designed for different habitats. The WG formulation is stored as a dry product and mixed into water for spraying as a liquid onto open, non-vegetated waters. The CG formulation is a dry granule and is applied to habitats with emergent vegetation, such as rice or wetlands. The granule is sprayed or thrown into the air, falls through the vegetation, and delivers the active ingredient directly to the water's surface. Both WG and CG formulations can be applied using a number of application methods and devices [47]. The volume of product required to treat a similar area is higher for CG than for WG due to differences in the international toxic units (ITU) per mg [13]. This has implications for shipping, transportation, and storage costs, which were all considered in this analysis. All application rates and product use costed here are based on published data [27].

### Mapping

Maps of the intervention areas are essential for programme planning and implementation. In Dar es Salaam we used the actual costs of purchasing aerial photographs and developing the maps from the UMCP [34]. In Vihiga District we included the cost of purchasing detailed maps of the area and staff time and equipment (GPS units) used to develop maps to guide larvicide application. In Mbita it was not necessary to include the cost of purchasing maps as satellite images of sufficient resolution can be downloaded free of charge from the internet (e.g. Google Earth). GPS units and staff time was included for the development of maps to guide applications.

### Monitoring and evaluation

A monitoring and evaluation (M&E) component must be an integral part in an operational vector control programme to document progress towards achievement of goals and for data-driven decision making on all levels of the programme [26,48]. LSM programmes need to monitor that: (1) the larvicide works (i.e. larvae dead after 24 hours), (2) LCPs are in the field applying larvicide according to their schedule, and (3) adult vector densities are significantly reduced compared to baseline and are kept at a low level throughout the year. Consequently, spot check larval habitat surveillance for monitoring product efficiency and staff performance and adult mosquito collections were included in the costing of the programmes. M&E strategies differed in the settings due to the differences in vector ecology, scale of the programmes and programme design. The impact of the intervention on the disease can be assessed through government health records in conjunction with the monitoring of other disease control efforts and was not costed as part of the LSM programme.

### Scenario analysis

Following development of the spreadsheet model for each intervention setting, cost implications of two scenarios using the alternative formulations of VectoBac^® ^were evaluated for Vihiga District (weekly application for four months) and Mbita Division (weekly application all year round).

Scenario 1 - use of WG formulation for liquid application;

Scenario 2 - use of CG formulation for hand application.

The type of formulation used affects product storage, associated equipment and shipping costs. The implications of changes in the larvicide product price in both of these scenarios were also examined. In Dar es Salaam larvicide product use was based on actual usage of a combination of WG and CG products, therefore a scenario analysis was not carried out however we did look at the implications of changes in larvicide product prices.

## Results

### Dar es Salaam, Tanzania

#### Model programme structure, staffing and training

The proposed programme structure for a LSM programme covering 15 city wards (5 per municipality) is shown in Figure 1. Table 2 lists the staff required and their responsibility in the programme. The programme is overseen by a programme manager who is directly responsible to the City Medical Officer and the NMCP. The manager is assisted in the daily work by two surveillance officers. Each of the three municipalities in Dar es Salaam has a malaria control coordinator that will ensure quality work implemented on ward level. Two inspectors per municipality will implement quality control and help with training activities. Each ward will have a supervisor overseeing the work of community-based resource persons (CORPs) involved in larval surveillance and larviciding. Two larval surveillance CORPs per ward will be responsible for habitat spot checks to ensure that larval habitats are treated in a timely manner and the larvicide is working. The number of LCPs was calculated based on the area one person can cover per week (0.2-1.5 km2) [34]. Technical assistance from an academic partner was assumed to be required for the equivalent of three months per annum. Support staff included three full time drivers for transporting staff and equipment, an administrator and general hand.

**Table 2:** Staff structure of model LSM program for 15 city wards of Dar es Salaam

Administrative level	Staff	Time or number of staff required	Role & responsibility
**PART TIME STAFF CONTRIBUTION TO PROGRAM**			

**International**	LSM expert	3 months/year	Advice on technical and programme management. Capacity building, support and trouble shooting.
**National**	Director of NMCP	1 hour/week	Approval of programme, reading progress reports, site visits.
	Procurement Officer	1 week/year	Central procurement of larvicide and equipment
**Regional**	City Medical Officer	1 week/year	Approval of programme, reading progress reports, signing documents, site visits.

**FULL TIME PROGRAM STAFF**			

**Regional**	City Program Manager	1	Day to day programme management, financial management. Recruiting and training Divisional Heads
	City Surveillance Officer	2	Assisting programme manager in field supervision of activities, training, community sensitization, supervision of adult vector monitoring, reports.
	Adult mosquito surveillance officer	1	Laboratory identification of adult mosquitoes
	City Mapping Officer	1*	Mapping of target area
	Mapping Technician	1*	Mapping of target area
	Driver	3	Transporting staff and equipment
	Administrator	1	Support to programme management and finance
	General hand	1	Support to office staff
**Municipal/** **District**	Municipal Coordinator	3	Program oversight at municipal level, review of data sheets, report writing
	Municipal Inspectors	6	Supervision and training of field staff, weekly reports.
**Ward**	Ward Supervisor	15	Supervision of larval survey CORPs and LCPs, distribution of larvicides, collation of field data, weekly reports
**TCU**	Larval Surveillance	30	Larval Habitat Spot Checks, reporting to supervisor.
	Larval Control	89	Treating breeding sites with larvicide, reporting to supervisor, collecting larvicide from store

* These salaries are treated as a capital cost because the detailed mapping is only required once.

It was assumed that the city and municipal level staff would receive specific training and support as required by the external technical adviser. These would then train other team members with support from the external technical adviser if required, e.g. inspectors and supervisors would train the LCPs. It was estimated that 135 LCPs and surveillance CORPs would need to be trained to provide sufficient coverage for the programme and allow for some attrition during training. LCPs would be trained prior to official recruitment and would, therefore, be paid a daily wage for training plus an allowance for transportation and lunch. Four six-day training sessions were calculated to be needed and a training room would need to be rented. Training would need to be repeated on an annual basis to account for attrition of staff and to adapt and improve programme delivery.

#### Larvicide, protective clothing, and application equipment

The quantity of larvicide required for 15 city wards was based on the actual use of VectoBac^® ^(CG and WG formulations) per km^2 ^from the three UMCP intervention wards in 2006. Calculations were based on the assumption that the CG formulation would be used for the majority of habitats since many of the aquatic sites are densely vegetated and can only be penetrated by granule. Furthermore, hand application is less prone to technical problems and was, therefore, chosen for the application of larvicides with CORPs in Dar es Salaam [26]. Nevertheless, large open water bodies can be more efficiently treated using liquid application with knapsack sprayers and therefore a budget is required for smaller quantities of WG formulations and application equipment. However, in order to enable comparison between similar programmes in the different locations the programme were all costed using CG formulation. As CG is the more expensive product this will if anything slightly increase the estimated cost of the programme. However the cost of equipment (30 Hudson spray pumps) needed to deliver the WG formulation was included as well as granule blowers (6) to allow treatment of large water surface areas with CG. More detail on product properties and decision-making processes for LSM programmes can be found elsewhere [26,27,49].

The intervention consisted of weekly treatment of all open (as opposed to closed, underground water e.g. septic tanks, latrines) water bodies within the 15 wards. Application and larval monitoring equipment requirements, including protective clothing were calculated according to the number of larval surveillance CORPs and LCPs employed. Itemized recurrent unit costs, useful life and capital costs are provided in Additional file 2.

#### Operations costs and overheads

Office and storage space requirements were calculated according to the number of staff and the volume of product and equipment that would be required at city and municipal levels. Overheads, such as utilities, insurance, and security, were considered along with other office support requirements, such as stationary, printing, photocopying, and communication (e.g. internet, telephone, and mobile telephones). Equipment, such as computers, printers, and global positioning system (GPS) units for mapping, was also included.

#### Transport and vehicles

Two project vehicles were required by the UMCP programme manager and the city malaria surveillance officers. The vehicles were also needed to transport the larvicide and equipment to the municipal areas. The municipal malaria control coordinators and inspectors used motorbikes to coordinate and supervise additional wards. Insurance, servicing, and fuel requirements were calculated based on UMCP data. Ward supervisors were not given vehicles, but instead were provided with a transportation allowance so they could use public transport around their area of responsibility and for staff meetings.

#### Adult mosquito monitoring

At the time of costing this programme, no traps were available to monitor the low density of vectors in the urban setting [26]. Therefore, human-landing catches were included as monitoring and evaluation tool here since this was the standard collection technique at the time. Nevertheless, it is expected that this tool will be replaced in the UMCP in the near future with the Ifakara Tent Trap which will be safer for the human bait, more efficient and cheaper than human-landing catches [50,51]. Allowances to community members for taking part in human landing catch surveys were calculated by assuming that there would be one person per *mitaa *(neighbourhood; there were 67 in the target area) for one night per week. Field equipment to collect mosquitoes (e.g. cups, gauzes and cool boxes), a microscope, light source, and adult surveillance officer/laboratory technicians' time were also included. Full details of the assumptions regarding recurrent and capital inputs and unit costs are provided in Additional File 2.

#### Dar es Salaam costing results

Table 3 shows the summary of the costing. The total annual cost of the programme was estimated at US$559, 476 to cover 592, 338 people located over 58 km2. Consequently, we estimate the cost per person protected to be US$0.94. Recurrent costs comprise 97% of the total programme costs. Cost, insurance, and freight (CIF) of the larvicide and protective clothing comprise 34% and programme staff salaries 31% of the total costs. Community sensitization (i.e. community education and briefing on programme activities) has a relatively high proportion of costs (9%) compared to the other programmes costed. Staff of the UMCP emphasized the importance of such communication tools in the urban setting. The printing and distribution of leaflets to households is crucial to ensure access to plots on treatment days. Taking the lowest and highest values of the published prices for VectoBac^® ^altered the cost per person protected to US$0.90 and US$0.98 respectively. At the lowest and highest prices for each product, the total programme costs are US$530, 866 and US$578, 262, respectively.

**Table 3:** LSM in urban Dar es Salaam: Financial and economic costs for 15 city wards (in US$ 2006 at midpoint larvicide price)

Cost category	Pre-implementation Costs (Y0) Total:		Implementation Year Costs (Y1) Total:		Average Annual Costs:		Proportion of Total Average:	
	Financial Cost	Economic Cost	Financial Cost	Economic Cost	Financial Cost	Economic Cost	Annual Financial Cost	Annual Economic Cost
**RECURRENT COSTS**								

International staff costs	30028.0	**30028.0**	30028.0	**30028.0**	33781.5	**33952.2**	0.05	**0.06**
NMCP/MoH staff costs	0.0	**46.1**	0.0	**46.1**	0.0	**52.1**	0	**0**
Program staff salaries	40349.5	**34801.5**	193569.0	**166953.2**	198612.6	**171501.2**	0.3	**0.31**
Larvicide (CIF) protective clothes	1500.0	**1500.0**	237771.2	**191480.8**	237958.7	**191676.8**	0.36	**0.34**
Staff Training	0.0	**0.0**	4724.9	**3779.9**	4724.9	**3779.9**	0.01	**0.01**
Community sensitization	0.0	**0.0**	66083.1	**52866.5**	66083.1	**52866.5**	0.1	**0.09**
Operations costs and overheads	39743.1	**31794.5**	46880.1	**37504.1**	51848.0	**41659.1**	0.08	**0.07**
Transport	11456.8	**9165.5**	37279.8	**30430.2**	38711.9	**31628.0**	0.06	**0.06**
Adult mosquito monitoring	17772.5	**15297.7**	17772.5	**15297.7**	19994.0	**17296.8**	0.03	**0.03**

**CAPITAL COSTS**								

Mapping area/breeding sites	965.9	**1039.5**	965.9	**1039.5**	1086.6	**1175.3**	0	**0**
Storage space and equipment	0.0	**0.0**	375.4	**295.4**	375.4	**295.4**	0	**0**
Vehicles	4664.7	**4074.0**	8396.4	**7239.8**	8979.5	**7772.2**	0.01	**0.01**
Spray pumps	1807.9	**1578.9**	1807.9	**1578.9**	2033.9	**1785.3**	0	**0**
Computers and other equipment	4090.0	**3466.8**	4090.0	**3466.8**	4601.3	**3919.8**	0.01	**0.01**
Adult monitoring equipment	400.0	**349.3**	400.0	**69.9**	450.0	**115.5**	0	**0**

SUBTOTAL RECURRENT COSTS	140850.0	**122633.2**	634108.4	**528386.4**	651714.7	**544412.7**	0.97	**0.97**

SUBTOTAL CAPITAL COSTS	11928.5	**10508.5**	16035.6	**13690.3**	17526.7	**15063.6**	0.03	**0.03**

TOTAL COSTS OF PROGRAM	152778.4	**133141.7**	650144.0	**542076.7**	669241.4	**559476.3**	1	**1**

COST PER PERSON PROTECTED	0.26	**0.22**	1.1	**0.92**	1.13	**0.94**		

### Vihiga District, western Kenya Highlands

#### Model programme structure, staffing and training

To plan the intervention, the district was divided into small manageable units. Because of the nature of the intervention and the topography of the district, these units are based on ecological rather than administrative zones. In a previous LSM project that informed this model structure, intervention areas were defined as ecological valleys (for details see [28]). Six valleys in Vihiga District correspond to the equivalent of approximately one sub-location (a Kenyan administrative unit below the location level). It was, therefore, generously estimated that throughout the whole district, each sub-location would contain ten ecological valleys. Using this unit and based on experience from the research project, we estimated the amount of resources that would be required to treat all breeding sites in the valley bottom and up the sides of the valleys at weekly intervals. Given the seasonal nature of peak vector densities in Vihiga District and increasing coverage with LLINs year round larviciding is not necessary [28]. The programme was developed around an intervention period beginning in February and ending in May. This 16-week period begins at the end of the dry season and continues throughout the long rainy season.

Based on experience, it was assumed that one person could treat all breeding sites within a valley in one day, and each LCP was given responsibility for three valleys. LCPs would be recruited from the local community thus maximizing local knowledge of potential sites, increasing local acceptance for the programme, and contributing to the local economy and LSM programme sustainability. This approach also has the advantage of reducing the need to transport LCPs around the district, thus minimizing transport costs. Although the intervention was assumed to last 16 weeks, costs were generously estimated based on employing LCPs for 18 weeks, not including training time which is captured under staff training. Therefore, it was estimated that 367 LCPs would be required in the whole Vihiga District. LCPs would be paid a daily wage for three days per week, plus one day's transportation allowance for collecting the larvicide from the divisional level storage location (see below). Support staff included a year round full time driver and an administrative assistant for the six month intervention phase.

LCPs were divided into 26 teams based on the number of locations in the district. Each team of LCPs would be managed by a supervisor who would be responsible for overseeing an average of 14 LCPs. It was assumed that supervisors would also be from the location which they managed and that they would take responsibility for working with the community to select LCPs. Given that the supervisors would need to be trained prior to recruiting the LCPs, it was assumed that they would be employed and paid a monthly wage for six months.

Six divisional heads (recruited and trained during the pre-intervention phase) would be responsible for managing and supervising activities within their respective divisions. Divisional heads would be full time staff paid an annual salary in order to prepare for the intervention phase, manage it, and then participate in evaluation and modification of the programme before the subsequent year's intervention. A programme manager would also be paid a full time annual salary to manage and oversee the programme. The team would be supported by a driver throughout the year and an administrative assistant employed for six months a year. It was also assumed that international experts would provide support to the programme for five months a year (some remote and some on site), although such high commitment would only be required at the start up of a new programme in the first 1 1/2 years. All individuals described above would need to be recruited by the programme and would spend all of their time during employment working on the larval control intervention. The opportunity cost of existing MoH employees, who would need to allocate some of their time to the programme, were also considered implicitly assuming the spare capacity of these individuals. The level of involvement that would be required from the Director of the NMCP, the NMCP entomologist, central level procurement officer, provincial level malaria control officer, district level public health officer, and district medical officer was estimated. Table 4 provides a summary of the personnel requirements for new project staff and existing MoH staff who would contribute a portion of their time to the programme.

**Table 4:** Staff structure of model LSM program for Vihiga District, Kenya

Administrative level	Number of staff		Time contributed/ period employed per year	Role & responsibility
**Part time staff contribution to program**				

**International**	LSM expert	1	5 months	Advice on technical and programme management. Capacity building, support and trouble shooting.
**National**	Director NMCP	1	1 week	Approval of programme, reading progress reports, signing documents, site visits.
	Entomologist	1	1 week	Reading progress reports, technical advice, site visits.
	Procurement Officer	1	1 week	Central procurement of larvicide and equipment
**Provincial**	Malaria Control Officer	1	1 week	Engagement in stakeholder meetings. Site visits with NMCP staff.
**District**	Public Health Officer	1	1 hour/week	Involvement in weekly management meetings.
	Medical Officer	1	2.5 weeks	Involvement in weekly management meetings, sensitization of District health staff.

**Full time program staff**				

**District**	Program manager	1	12 months	Day to day programme management, financial management. Recruiting and training Divisional Heads.
	Driver	1	12 months	Transport of programme manager, transport of equipment and supplies
	Admin. assistant	1	6 months	Administrative duties during intervention and for one month during pre and post intervention phase
**Division**	Divisional Heads	6	12 months	Field work management, quality control, adult mosquito monitoring, reporting to programme manager. Evaluation and planning subsequent year's intervention. Training of Supervisors and LCP.
**Location**	Supervisors	26	6 months	Recruitment and supervision of LCP, larval habitat spot checks.
**Valleys**	LCP	367	18 weeks	Treating breeding sites with larvicide, reporting to supervisors, collecting larvicide from divisional store.

It was assumed that the programme manager would be a biological science graduate and be trained by the international expert in programme management. The programme manager and international expert would then recruit and train the divisional heads. A one-week trip to visit the operational LSM programme in Dar es Salaam, Tanzania was also incorporated for the programme manager and divisional heads as part of in-service training. Once recruited, it was assumed that supervisors would be trained by divisional heads. Supervisors and divisional heads would then train the LCPs. It was assumed that 420 LCPs would be trained to provide sufficient coverage for the programme and allow for some attrition during training. LCPs would be trained prior to recruitment and would, therefore, be paid a daily wage for training, plus an allowance for transportation and lunch. It was calculated that 12 six-day training sessions would be needed with 35 LCPs per course. The assumption was made that training would take place in the intervention sites and in locations where communities usually meet (i.e. outdoor locations that do not require rental of space). Training would need to be repeated on an annual basis to account for staff attrition staff.

#### Larvicide product, protective clothing, and application equipment

The quantity of larvicide required was based on usage of VectoBac^® ^in a previous LSM project implemented in 3 valleys of Vihiga District [28]. Mean monthly usage was calculated from the three sites and multiplied by the number of valleys in the district to obtain the total amount required to treat the whole district. The implications of using the two alternative formulations in terms of transport, storage, and equipment requirements were captured as follows. If only WG formulation would be used 400 Hudson spray pumps would be required (one per LCP and some spares to account for loss through breakage and theft). CG granule is applied by hand and we included buckets or backpacks as carriage containers instead of sprayers. Other field equipment that is required independently from the formulation used e.g. protective clothing for LCPs, was itemized. For both scenarios, we considered shipping the product from the U.S. to Mombasa (including insurance), port clearance fees, and ground transport from Mombasa to Vihiga District, based on the required volume and weight of the product. Storage and distribution of the product once arrived at the district level was considered under operations costs and overheads (see below).

#### Operations costs and overheads

A furnished office would be needed all year at the district level, including insurance, utilities, and a security guard. The amount of storage space required was estimated to keep each of the alternative larvicide formulations and other equipment at the district level in secure metal containers. It was assumed that five additional furnished offices with storage space would be required at the divisional levels for six months each year. Insurance, utilities, and security guards for these offices were also included. A truck and driver would need to be hired for one day per month if WG formulation was used and two days per month if CG formulation was used during the four-month intervention period to transport the larvicide from the district to the divisional offices. Transport of the product from the divisional offices to the intervention valleys would be done on a weekly basis by LCPs, who would be given a travel allowance equivalent to one day's wage, to do this in whatever way they chose. Appropriate staff was allocated a mobile phone and monthly communication allowance to purchase phone credits for the duration of their employment. Computers, printers, GPS units, and Internet connectivity were also considered along with requisite software. Stationary, printing, and photocopying requirements were captured.

#### Transport and vehicles

A quote for a suitable project vehicle was obtained from a local supplier. Data on fuel, insurance, servicing, and repair requirements for a similar vehicle used in same area were obtained from Kenya Medical Research Institute (KEMRI) records. Divisional heads would each need a motorbike, including fuel, insurance, and maintenance. Given the relatively large distances to be covered by the LCPs, it was assumed that each would be furnished with a bicycle. This was treated as a recurrent cost because it was assumed that after one year, the bicycles would be given to the LCP. In return, the LCP would be responsible for maintenance and repair of the bicycles during the intervention and would have an incentive to take care of them.

#### Adult mosquito monitoring

Adult mosquito monitoring would be required year round in a stratified sample of approximately 117 houses within each division (approx. 1 collection site/km^2^) to monitor effectiveness of the programme during the intervention period. It was also considered necessary to maintain this monitoring throughout the year to ensure that the intervention was appropriately targeted in time. This activity would be carried out by the divisional heads using locally produced clay pots, which have been shown to provide effective outdoor catches for adult mosquitoes [52]. A microscope and light source, as well as other supplies, including ethanol, vials, Petri dishes, and dissecting kits, would be required for this activity. Mosquito identification would be done by the programme manager with assistance of Division heads. Full details of the assumptions regarding recurrent and capital inputs and unit costs are provided in the Additional File 3.

#### Vihiga District costing results

Table 5 shows the results of the costing considerations for Scenario 1 - WG use only. The cost per person protected using the WG larvicide formulation was US$0.79, and the total annual programme costs were estimated at US$480, 735 to cover 609, 324 people located over 563 km2. Recurrent costs make up 94% of total costs. A fifth of total cost is allocated to programme staff salaries. Operations costs and overheads contribute 13% of programme costs. CIF of the larvicide and protective clothing make up almost half (47%) of total programme costs.

**Table 5:** LSM in western Kenya highlands - Vihiga District: Scenario 1 - Financial and economic costs using VectoBac WG formulation (in US$ 2006 at midpoint larvicide price)

Cost category	Pre-implementation Costs (Y0) Total:		Implementation Year Costs (Y1) Total:		Average Annual Costs:		Proportion of Total Average:	
	Financial Cost	Economic Cost	Financial Cost	Economic Cost	Financial Cost	Economic Cost	Annual Financial Cost	Annual Economic Cost
**RECURRENT COSTS**								

International staff costs	23013.0	**23013.0**	34665.0	**34665.0**	37541.7	**37672.5**	0.07	**0.08**
NMCP/MoH staff costs	0.0	**0.0**	0.0	**826.3**	0.0	**826.3**	0	**0**
Programme staff salaries	11484.6	**9848.0**	110894.0	**95091.6**	112329.5	**96378.6**	0.22	**0.2**
Larvicide (CIF), protective cloth	0.0	**0.0**	230775.3	**226247.4**	230775.3	**226247.4**	0.44	**0.47**
Staff Training	0.0	**0.0**	12820.4	**10769.2**	12820.4	**10769.2**	0.02	**0.02**
Community sensitization	0.0	**0.0**	68.9	**57.8**	68.9	**57.8**	0	**0**
Operations costs and overheads	1985.5	**1667.8**	75984.8	**63827.3**	76233.0	**64045.2**	0.15	**0.13**
Transport	6324.8	**5312.9**	14081.8	**11828.7**	14872.4	**12523.1**	0.03	**0.03**
Adult mosquito monitoring	799.8	**671.8**	1599.6	**1343.7**	1699.6	**1431.5**	0	**0**

**CAPITAL COSTS**								

Maps	66.7	**83.8**	66.8	**83.8**	75.0	**94.8**	0	**0**
Vehicles	5095.1	**4636.2**	10190.3	**9272.5**	10827.2	**9878.4**	0.02	**0.02**
Spray pumps	0.0	**0.0**	20000.0	**18340.6**	20000.0	**18340.6**	0.04	**0.04**
Computers, other equipment	871.7	**774.9**	2330.0	**2072.4**	2439.0	**2173.6**	0	**0**
Adult monitoring equipment	200.0	**183.4**	400.0	**366.8**	425.0	**390.8**	0	**0**

SUBTOTAL RECURRENT COSTS	43, 607.8	**40, 513.6**	480, 889.8	**444, 656.9**	486, 340.7	**449, 951.4**	0.94	**0.94**

SUBTOTAL CAPITAL COSTS	6, 166.8	**5, 594.5**	32, 920.3	**30, 052.3**	33, 691.1	**30, 783.4**	0.06	**0.06**

TOTAL PROGRAM COST	49, 774.5	**46, 108.1**	513, 810.0	**474, 709.1**	520, 031.9	**480, 734.7**	1	1

COST PER PERSON PROTECTED	0.08	**0.08**	0.84	**0.78**	0.85	**0.79**		

Taking the lowest value of the published prices for VectoBac^® ^WG reduced the economic cost of the programme to US$437, 753 and the cost per person protected falls to $0.72. Taking the highest published price for VectBac^® ^increases the total programme costs to US$511, 306 and the costs per person protected increases by $0.05 to US$0.84.

Table 6 shows the results of the costing considerations for Scenario 2 - CG use only. At midpoint prices, the economic cost per person protected is US$1.50. The total programme costs are US$916, 908. In this scenario, CIF of the larvicide and protective clothing make up around two thirds of programme costs (67%). Taking the lowest value of CG prices reduced the cost per person to US$1.35 and the total programme costs to US$823, 668. Taking the highest published price increases the total programme costs to US$978, 130 and the cost per person protected increases to US$1.61.

**Table 6:** LSM in western Kenya highlands - Vihiga District: Scenario 2 - Financial and economic costs using VectoBac CG formulation (in US$ 2006 at midpoint larvicide price)

Cost category	Pre-implementation Costs (Y0) Total:		Implementation Year Costs (Y1) Total:		Average Annual Costs:		Proportion of Total Average:	
	Financial Cost	Economic Cost	Financial Cost	Economic Cost	Financial Cost	Economic Cost	Annual Financial Cost	Annual Economic Cost
**RECURRENT COSTS**								

International staff costs	23013.0	**23013.0**	34665.0	**34665.0**	37541.7	**37672.5**	0.04	**0.04**
NMCP/MoH staff costs	0.0	**0.0**	0.0	**60001.1**	0.0	**60001.1**	0	**0.07**
Programme staff salaries	11484.6	**9848.0**	110894.0	**95091.6**	112329.5	**96378.6**	0.12	**0.11**
Larvicide (CIF), protective cloth	0.0	**0.0**	625985.3	**616959.8**	625985.3	**616959.8**	0.69	**0.67**
Staff Training	0.0	**0.0**	12820.4	**10769.2**	12820.4	**10769.2**	0.01	**0.01**
Community sensitization	0.0	**0.0**	68.9	**57.8**	68.9	**57.8**	0	**0**
Operations costs and overheads	1985.5	**1667.8**	81493.1	**68454.2**	81741.3	**68672.1**	0.09	**0.07**
Transport	6324.8	**5312.9**	14081.8	**11828.7**	14872.4	**12523.1**	0.02	**0.01**
Adult mosquito monitoring	799.8	**671.8**	1599.6	**1343.7**	1699.6	**1431.5**	0	**0**

**CAPITAL COSTS**								

Maps	66.7	**83.8**	66.8	**83.8**	75.0	**94.8**	0	**0**
Vehicles	5095.1	**4636.2**	10190.3	**9272.5**	10827.2	**9878.4**	0.01	**0.01**
Spray pumps	0.0	**0.0**	0.0	**0.0**	0.0	**0.0**	0	**0**
Computers, other equipment	871.7	**774.9**	2330.0	**2072.4**	2439.0	**2173.6**	0	**0**
Adult monitoring equipment	200.0	**183.4**	400.0	**366.8**	425.0	**390.8**	0	**0**

SUBTOTAL RECURRENT COSTS	43, 607.8	**40, 513.6**	881, 608.1	**899, 171.1**	887, 059.0	**904, 465.6**	0.98	**0.99**

SUBTOTAL CAPITAL COSTS	6, 166.8	**5, 594.5**	12, 920.3	**11, 711.6**	13, 691.1	**12, 442.8**	0.02	**0.01**
TOTAL COSTS OF PROGRAM	49, 774.5	**46, 108.1**	894, 528.3	**910, 882.7**	900, 750.2	**916, 908.3**	1	**1**

COST PER PERSON PROTECTED	0.08	**0.08**	1.47	**1.49**	1.48	**1.5**		

### Mbita Division, Suba District, shores of Lake Victoria, western Kenya

#### Model programme structure, staffing and training

Given the perennial nature and heterogeneity of malaria transmission in Mbita Division and the predominance of people-made habitats, year round larviciding was assumed necessary to control malaria vectors in this location [14]. The amount of resources that would be required to treat all breeding sites in 140 km^2 ^of Mbita Division on a weekly basis, year round based was estimated based on data available from a previous small scale LSM project [27]. The model programme structure is shown in Figure 3.

One locally-recruited LCP would be required to treat all breeding sites in one km^2 ^each per day. Each LCP would therefore be given an area of five km^2 ^to treat each week. To cover the total area, 28 LCPs would be required. Supervisors would monitor the work of the LCPs and carry out larval spot checks. Three supervisors would be required and each would be responsible for nine to ten LCPs and approximately 47 km^2 ^of land. One programme manager would be required to oversee the programme, along with a driver. All staff would be employed year round and would spend all their time during employment working on the larval control intervention.

The opportunity costs of existing MoH employees who would need to allocate some of their time to the programme were also considered. The level of involvement that would be required from the provincial malaria control officer, district level public health officer, and DMO were estimated. International expert support was included for two months per year. Table 7 provides a summary of personnel requirements for both full time project staff and existing staff who would contribute a portion of their time to the programme.

**Table 7:** Staff structure of model LSM program for Mbita Division, western Kenya

Administrative level	Number of staff		Time contributed/ period employed per year	Role and responsibilities
**PART TIME STAFF CONTRIBUTION TO PROGRAM**				

**International**	LSM expert	1	2 months	Advice on technical and programme management. Capacity building, support and trouble shooting.
**Provincial**	Malaria Control Officer	1	1 week	Engagement in stakeholder meetings. Site visits with NMCP
**District**	Public Heath Officer	1	1 hour/week	Involvement in weekly management meetings.
	Medical Officer	1	2.5 weeks	Involvement in weekly management meetings, sensitization of District health staff.

**FULL TIME PROGRAM STAFF**				

**District**	Program manager	1	12 months	Day to day programme management, procurement, financial management. Recruiting and training Divisional supervisors
	Driver	1	12 months	Transport of program manager. Delivery of larvicide to supervisors/LCP.
**Division/** **LSM-Area**	Supervisors	3	12 months	Field work management, quality control, larval spot checks and adult collections, reporting to programme manager. Evaluation and planning subsequent year's intervention. Training of Supervisors and LCP Recruitment and supervision of LCP.
**5 km^2 ^LSM-Unit**	LCP	28	12 months	Larvicide application, recording and reporting to supervisors

Assumption was that the programme manager would be a postgraduate in biological sciences and be trained by the international expert in larval programme management. The programme manager and international expert would then recruit and train the supervisors. A one-week trip to visit an operational larval control programme in Tanzania for the programme manager and supervisors as part of in-service training was costed. Supervisors would train 35 LCPs, which would provide sufficient coverage for the programme and allow for some attrition during/after training. LCPs would be trained prior to recruitment and would, therefore, be paid a daily wage for training, plus an allowance for transportation and lunch. One six-day training session would be needed. Training would take place in the intervention sites and in locations where communities usually meet (i.e., outdoors locations that do not require rental of space). Training would need to be repeated on an annual basis to account for staff attrition.

#### Larvicide product, protective clothing, and application equipment

Mean monthly usage of VectoBac^® ^in kilograms per km2 of land and water surface area treated was calculated based on usage in a previous LSM project implemented in Mbita town for over two years[27]. The implications of using the two alternative formulations in terms of transport, storage, and equipment requirements were captured in a similar way as for Vihiga District. Protective clothing and other equipment requirements were calculated according to the number of LCPs operating. The product would be shipped from the U.S. to Mombassa (including insurance). Port clearance fees, and ground transport from Mombassa to Mbita, based on the required volume and weight of the product were costed.

#### Operations costs and overheads

Office and storage space rental at the divisional level was captured in the same way as for Vihiga District. Communication costs, including mobile phones, phone usage credit, and Internet access, were accounted for. Office equipment, including a computer, printer, stationary, and printing requirements, were also included.

#### Transport and vehicles

One project vehicle would be required, along with motorcycles for the supervisors and bicycles for the LCPs. Fuel and maintenance costs of the vehicles and motorcycles were included. As described for previous locations, LCPs were responsible for the maintenance and care of bicycles, which were again treated as recurrent cost items.

#### Adult mosquito monitoring

Adult mosquito monitoring would be required year round in a stratified sample of 152 houses (on average approximately 1 sampling site/km^2^) within the division. This activity would be carried out by the supervisors using locally produced clay pots [52]. A microscope and light source, as well as other supplies including ethanol, vials, Petri dishes, and dissecting kits, would be required for this activity, and subsequent mosquito identification would be carried out by the supervisors and programme manager.

#### Mbita Division costing results

The total financial and economic costs and cost per person are shown in Table 8 for scenario 1 (WG use only) and in Table 9 for scenario 2 (CG use only) both at mid-point prices. The cost per person protected using WG formulation for liquid application was US$1.94, and the total annual programme costs were estimated at US$107, 669 to cover 55, 558 people located over 211 km2. Using CG the cost per person protected was US$2.50, and the total programme cost was US$138, 866.

**Table 8:** LSM at shores of Lake Victoria in Mbita Division: Scenario 1 - Financial and economic costs using VectoBac WG formulation (in US$ 2006 at midpoint larvicide price)

Cost category	Pre-implementation Costs (Y0) Total:		Implementation Year Costs (Y1) Total:		Average Annual Costs:		Proportion of Total Average:	
	Financial Cost	Economic Cost	Financial Cost	Economic Cost	Financial Cost	Economic Cost	Annual Financial Cost	Annual Economic Cost
**RECURRENT COSTS**								

International staff costs	9516.0	**9516.0**	19032.0	**19032.0**	20221.5	**20275.6**	0.16	**0.18**
NMCP/MoH staff costs	0.0	**0.0**	344.3	**847.8**	344.3	**847.8**	0	**0.01**
Programme staff salaries	14426.0	**12370.3**	39488.5	**33861.4**	41291.8	**35478.0**	0.33	**0.31**
Larvicide (CIF), protective cloth	0.0	**0.0**	22601.9	**21867.7**	22601.9	**21867.7**	0.18	**0.19**
Staff Training	0.0	**0.0**	2901.5	**2437.2**	2901.5	**2437.2**	0.02	**0.02**
Community sensitization	0.0	**0.0**	144.6	**121.5**	144.6	**121.5**	0	**0**
Operations Costs and Overheads	5087.2	**4273.3**	6739.7	**5661.4**	7375.6	**6219.8**	0.06	**0.05**
Transport	6229.1	**5232.5**	8696.8	**7305.3**	9475.5	**7989.1**	0.08	**0.07**
Adult mosquito monitoring	415.3	**348.9**	415.3	**348.9**	467.2	**394.5**	0	**0**

**CAPITAL COSTS**								

Vehicles	8813.2	**8045.8**	8813.2	**8045.8**	9914.8	**9097.3**	0.08	**0.08**
Spray pumps	0.0	**0.0**	1500.0	**1375.6**	1500.0	**1375.6**	0.01	**0.01**
Computers, other equipment	1136.7	**1009.4**	1147.0	**1018.4**	1289.1	**1150.4**	0.01	**0.01**
Adult monitoring equipment	400.0	**366.8**	400.0	**366.8**	450.0	**414.8**	0	**0**

SUBTOTAL RECURRENT COSTS	35673.7	**31740.9**	100364.7	**91483.2**	104823.9	**95631.2**	0.83	**0.83**

SUBTOTAL CAPITAL COSTS	10349.9	**9422.0**	11860.2	**10806.6**	13153.9	**12037.9**	0.1	**0.1**

TOTAL COSTS OF PROGRAM	46023.6	**41162.9**	112224.8	**102289.8**	117977.8	**107669.2**	1.00	**1.00**

COST PER PERSON PROTECTED	0.83	**0.74**	2.02	**1.84**	**2.12**	**1.94**		

**Table 9:** LSM at shores of Lake Victoria in Mbita Division: Scenario 2 - Financial and economic costs using VectoBac CG formulation (in US$ 2006 at midpoint larvicide price)

Cost category	Pre-implementation Costs (Y0) Total:		Implementation Year Costs (Y1) Total:		Average Annual Costs:		Proportion of Total Average:	
	Financial Cost	Economic Cost	Financial Cost	Economic Cost	Financial Cost	Economic Cost	Annual Financial Cost	Annual Economic Cost
**RECURRENT COSTS**								

International staff costs	9516.0	**9516.0**	19032.0	**19032.0**	20221.5	**20275.6**	0.14	**0.15**
NMCP/MoH staff costs	0.0	**0.0**	344.3	**847.8**	344.3	**847.8**	0	**0.01**
Programme staff salaries	14426.0	**12370.3**	39488.5	**33861.4**	41291.8	**35478.0**	0.28	**0.26**
Larvicide (CIF), protective cloth	0.0	**0.0**	55583.6	**54440.5**	55583.6	**54440.5**	0.37	**0.39**
Staff Training	0.0	**0.0**	2901.5	**2437.2**	2901.5	**2437.2**	0.02	**0.02**
Community sensitization	0.0	**0.0**	144.6	**121.5**	144.6	**121.5**	0	**0**
Operations Costs and Overheads	5087.2	**4273.3**	6739.7	**5661.4**	7375.6	**6219.8**	0.05	**0.04**
Transport	6229.1	**5232.5**	8696.8	**7305.3**	9475.5	**7989.1**	0.06	**0.06**
Adult mosquito monitoring	415.3	**348.9**	415.3	**348.9**	467.2	**394.5**	0	**0**

**CAPITAL COSTS**								

Vehicles	8813.2	**8045.8**	8813.2	**8045.8**	9914.8	**9097.3**	0.07	**0.07**
Spray pumps	0.0	**0.0**	0.0	**0.0**	0.0	**0.0**	0	**0**
Computers and other equipment	1136.7	**1009.4**	1147.0	**1018.4**	1289.1	**1150.4**	0.01	**0.01**
Adult monitoring equipment	400.0	**366.8**	400.0	**366.8**	450.0	**414.8**	0	**0**

SUBTOTAL RECURRENT COSTS	35673.7	**31740.9**	133346.3	**124056.0**	137805.5	**128204.0**	0.92	**0.92**

SUBTOTAL CAPITAL COSTS	10349.9	**9422.0**	10360.2	**9431.1**	11653.9	**10662.4**	0.08	**0.08**

TOTAL COSTS OF PROGRAM	46023.6	**41162.9**	143706.5	**133487.0**	149459.5	**138866.4**	1	**1**

COST PER PERSON PROTECTED	0.83	**0.74**	2.59	**2.4**	**2.69**	**2.5**		

The proportion of total programme costs allocated to CIF of the larvicide and protective clothing ranges from 19% if WG is used to 39% if CG is used. Staff salaries are a significant contributor to programme costs and range from 26% (CG usage) to 31% (WG usage).

Taking the lowest value of WG prices reduced the cost per person to US$1.88 and the total programme costs to US$104, 266. Taking the highest value of WG prices increases the cost per person to US$1.98 and total programme costs to US$110, 090. Taking the lowest value of CG prices reduced the cost per person to US$2.37 and the total programme costs to US$131, 484. Taking the highest published price increases the total programme costs to US$143, 714 and the cost per person protected increases to US$2.59.

Full details of the assumptions regarding recurrent and capital inputs and unit costs are provided in the Additional File 4.

## Discussion

The analyses presented here shows that cost of LSM using microbial larvicides varies in different eco-epidemiological settings in East Africa. The results show that for programmes using the same formulation (CG) larviciding costs the least in urban Dar es Salaam (US$0.94), 60% more in the highly populated rural highlands of Vihiga District (US$1.50) in a targeted approach and the most (US$2.50) in Mbita Division where population density is lower and year round application is required. However, these costs are reduced substantially if the alternative formulation (WG) is used; in Vihiga this would reduce costs to US$0.79 and in Mbita Division to US$1.94. (See Table 10 for summary results).

**Table 10:** Summary of Costing Results (US$ 2006) from Dar es Salaam, Vihiga District, and Mbita Division

Location (population protected)	Scenario	Cost type	Price of Larvicide					
			Low		Mid		High	
			
			Total cost	Cost/person	Total cost	Cost/person	Total cost	Cost/person
**Dar es Salaam (592, 338)**	**CG**	**Financial**	632, 048	**1.07**	669, 241	**1.13**	693, 663	**1.17**
		**Economic**	530, 866	**0.90**	559, 476	**0.94**	578, 262	**0.98**

**Vihiga (609, 324)**	**WG**	**Financial**	477, 050	**0.78**	520, 032	**0.85**	550, 603	**0.90**
		**Economic**	437, 753	**0.72**	480, 735	**0.79**	511, 306	**0.84**
	
	**CG**	**Financial**	807, 510	**1.33**	900, 750	**1.48**	961, 972	**1.58**
		**Economic**	823, 668	**1.35**	916, 908	**1.50**	978, 130	**1.61**

**Mbita (55, 558)**	**WG**	**Financial**	114, 574	**2.06**	117, 978	**2.12**	120, 398	**2.17**
		**Economic**	104, 266	**1.88**	107, 669	**1.94**	110, 090	**1.98**
	
	**CG**	**Financial**	142, 077	**2.56**	149, 459	**2.69**	154, 307	**2.78**
		**Economic**	131, 484	**2.37**	138, 866	**2.50**	143, 714	**2.59**

In reality, as in the Dar es Salaam programme, a combination of formulations are likely to be required depending on the aquatic habitat types and ecology, implying that costs will fall between these two data points. Hence the appropriate product formulation used will influence costs. Habitats that have less vegetation will allow for greater use of the WG formulation which will generally result in less expensive programmes than those that require the CG formulation, even taking into account additional application equipment needs, such as spray pumps. Ultimately, product formulation decisions should be based on the local vector ecology and habitats as well as practical and operational considerations including operator and community preferences. Discussions with staff involved in various LSM projects revealed that the hand application of CG granule is often preferred over knapsack sprayer application [26,49]. It is important to note that the lowest cost product will not necessarily result in the most cost effective programme, because of the range of other factors that must be addressed in each programme.

Another key factor influencing cost is population density; because the intervention targets spatial areas, the higher the population density the lower the cost per person protected (other things being equal). Increasing the population protected will spread programme costs, especially fixed overheads, over a larger number of people and hence reduce per capita costs. Population density also has an impact on the number of individuals protected through application of larvicides in a given area.

The nature of malaria transmission and risk (influenced by other malaria control interventions) also has an impact on cost. The ability to target larvicididing in space (as in Vihiga and Mbita) and in time (Vihiga) provides opportunities to increase the efficiency of operations and thus reduce cost per person protected. In the highland area of Vihiga District, the programme costs are reduced (relative to the other programmes) because we hypothesized that treatment of breeding sites is not required year round to interrupt transmission which is seasonal.

Recently, a study was designed to investigate whether LSM in these sites can reduce malaria infections [28]. Evidence was provided of the impact of a double vector control intervention directed at both larval and adult mosquitoes. Vector control with microbial larvicides and LLINs combined, resulted in a two-fold reduction in new malaria infections compared with nets alone indicating that the addition of LSM to LLIN programmes can provide substantial additional protection against malaria parasites. Furthermore, the study indicated that vector densities only peaked during the main transmission season (March to June), when bednet coverage was high [28]. It is, therefore, likely that targeting larval control for a brief period at the end of the dry season and beginning of the long rainy season could be as efficient at controlling malaria as continual application throughout the year. This allows some programme staff to be employed on a seasonal basis, thus reducing costs. It is important to note that adequate monitoring does need to take place year round in such a programme to ensure the intervention is targeted optimally in relation to vector density and malaria transmission. However, the option of targeting interventions to the specific timing of the malaria transmission season presents options for reducing cost, which must be balanced against required surveillance costs (around US$17, 000 in Dar es Salaam, where surveillance is relatively intensive). This is analogous to IRS in areas of seasonal or epidemic transmission where the cost effectiveness of the intervention has been shown to vary with timing in relation to the transmission season [53,54]. Research is underway to identify appropriate targeting strategies including LSM [55-57].

Programme structure will also drive costs, and in Dar es Salaam the management structure and number of employees is higher, reflecting the operational realities of working in an urban environment. However, the additional costs of this programme structure are moderated by higher population density. In Mbita Division, the programme staff structure is minimal, however, the number of people protected is also lower, which means that fixed costs, such as international expert support and programme staff salaries, contribute a larger portion to per capita costs. Costs per person might decrease if the entire Suba district would be targeted for LSM since this could still be done with one programme manager and the estimated amount of external support. Identifying the optimal operational scale and management structure for potential LSM programmes will be critical area where vector control programmes may need support.

A further consideration for costs is the inclusion of the control of nuisance mosquitoes (*Culex *spp.) in a programme. This was outside the scope of this project but will be a question raised especially in the urban context where, like in Dar es Salaam, culicine mosquitoes are responsible for over 100 bites per exposed person per night [26]. While targeting the interventions only at potential *Anopheles *breeding sites can reduce costs, it may not improve cost effectiveness. Access to breeding sites on private property is essential and so a withdrawal of communities' support may reduce effectiveness [58-60]. The control of nuisance mosquitoes in Dar es Salaam to date remained unsatisfactory, the overall culicine densities remained high in the intervention wards due to the large number of closed habitats like pit latrines, soakage pits, septic tanks and water storage tanks, which were not included in the weekly larvicide applications [26]. Efficient strategies including the implementation of environmental modifications need to be developed and costed to address the nuisance biting problem in urban areas, especially since *Culex *and *Aedes *mosquitoes are also important vectors of diseases, such as lymphatic filariasis, encephalitis, dengue and chikungunya in many parts of the world [61].

To date very little information is available for the costs of LSM in contemporary Africa. A basic cost estimation was attempted by Fillinger and Lindsay estimating that the cost of protecting the human population in the study area of Mbita town was approximately US$0.90 per person protected [27]. Our results for Mbita Division are higher for a number of reasons. First, we considered all programme costs, rather than only direct expenditure on consumables and salaries. We have included a complete staffing structure required to operate a programme at scale, including external technical assistance, vehicles, office space and overheads. Second, variation in population density is very important. Fillinger and Lindsay protected 8, 000 people in an urbanized setting of approximately 4.5 km^2 ^in Mbita town [27]. Here costs are estimated for the whole of Mbita Division, where average population density is three times lower.

The costing methods used in this analysis are standard and consistent with those used in other economic evaluations of malaria control interventions [62-64]. Importantly, this paper enables the larval control costs be compared to other malaria and health interventions, such as IRS and ITNs [53]. However, the output indicator used in this report is person protected per year. This indicator was chosen because at the time of the analysis there was insufficient data on the epidemiological impact of larval control to estimate the additional benefits (e.g. malaria cases prevented, malaria-related deaths averted, or disability adjusted life years [DALYs] saved) of using larval control in conjunction with other malaria vector control interventions. Forthcoming evidence on the effectiveness of LSM on malaria prevention can be incorporated into these costing models.

## Conclusions

The integration of LSM into ongoing malaria control programmes is likely to be most effective where transmission is moderate or low and where mosquito breeding sites are contained and well-defined [14,27,28,31]. These include, but are not restricted to, highland areas, desert-fringe areas, urban settings and areas prone to epidemics. The current success of ITN programmes to reduce malaria transmission will result in far more areas with low and focal malaria transmission; representing a great opportunity for IVM programmes, including LSM, to maintain hard won gains and aim for even further reductions. Cost implications are of major importance for deciding which interventions to use in an IVM programme, yet these data are lacking. This is particularly important since the World Health Organisation is encouraging the adoption of IVM for the control of malaria and other vector borne diseases [4]. This economic analysis bears out the previously highlighted suggestion that focusing LSM efforts in areas of high population density where spatial and/or temporal targeting is possible offers a low cost intervention in terms of cost per person protected per year. Nevertheless, such settings are not only found in urban areas.

During the course of this analysis, three potential sites for larval control programmes of different sizes and environmental settings were visited. In each, an operational larval control programme or research study was currently operating. The individuals consulted during development of these costing models described, based on their local experiences, the needs for a large-scale operational LSM programmes that would be appropriate for each setting; where needed, specific programmatic challenges were addressed with locally appropriate solutions. While careful consideration of the ecological and operational conditions in each setting is required, the cost models developed here will facilitate cost implication evaluation for alternative programme approaches and structures.

In appropriate settings and based on the current entomological evidence, LSM is an attractive malaria control intervention in terms of cost. The cost per person protected by larval control in this analysis ranged from US$0.79 to US$2.50, which is comparable with other malaria interventions. For example the cost of IRS ranges from US$0.88-4.94 per person protected (US$2000), the cost per treated net year for conventional ITNs was found to range from US$1.21-6.05 and for LLINs US$1.38-1.90 (2005 US$) [54,65]. Analyses such as the Disease Control Priorities Project have described malaria control interventions as among the most cost effective health interventions and have recommended investment in malaria control as an essential development and poverty reduction strategy [66]. The analysis presented here addresses one of the major concerns hampering the advocacy of LSM to date, the perception that this intervention is very expensive. The presented costing analysis indicates that LSM is within the cost range of other interventions that have been described as among the most cost effective. Therefore, larval control can be a complementary tool in the malaria control effort in selected settings. Malaria control programme managers and other decision makers in national and international organizations should consider LSM as part of an IVM strategy, in conjunction with ITNs and/or IRS. However, LSM should not be considered as a replacement for other vector control methods or as a stand-alone intervention.

Once further evidence is available with which to estimate the additional health benefits of using LSM in conjunction with other vector control interventions, it will be possible to complete an analysis of the comparative cost effectiveness of various combinations of IRS, ITNs, and LSM. Other larval control products are available on the market, including insect growth regulators (IGR), synthetic organic chemicals, and microbials other than those considered in this paper. Investigation into the cost and programmatic implications of these products and/or formulations should be considered (e.g., considering the costs and risks associated with insecticide resistance development, impacts on non-target organisms, and worker safety).

## Competing interests

This work was partially supported by Valent BioSciences Corp., the commercial manufacturer of the microbial larvicide costed. Nevertheless, none of the funders of this work had any role in the analysis or interpretation of the results, nor in the drafting of the manuscript.

## Authors' contributions

EW and UF collated all the data necessary for analyses, UF advised on the design of the model programmes and ingredients and EW did the costing analyses. Both authors jointly wrote the manuscript, read and approved the final version.

## Supplementary Material

Additional file 1 **Larvicide product price, exchange rates and annulization factors**. The document presents tables with the product prices used for the costing, exchange rates used for currency conversion and the annulization factor and discount rates used for capital costs. Click here for file

Additional file 2 **Dar es Salaam: Recurrent and capital unit costs**. The file shows two tables itemizing the recurrent cost units and the capital cost units on which the economic costing is based. Click here for file

Additional file 3 **Vihiga District: Recurrent and capital unit costs**. The file shows two tables itemizing the recurrent cost units and the capital cost units on which the economic costing is based. Click here for file

Additional file 4 **Mbita Division: Recurrent and capital unit costs**. The file shows two tables itemizing the recurrent cost units and the capital cost units on which the economic costing is based. Click here for file
