# Supplementary material for: Large-scale use of mosquito larval source management for malaria control in Africa: a cost analysis
Source: Malar J. 2011 Nov 8;10:338. doi: 10.1186/1475-2875-10-338 (PMC3233614; doi:10.1186/1475-2875-10-338)
Supplement: Additional file 1 — Larvicide product price, exchange rates and annulization factors. The document presents tables with the product prices used for the costing, exchange rates used for currency conversion and the annulization factor and discount rates used for capital costs. [file 1475-2875-10-338-S1.PDF]

Additional file 1:
Larvicide product price, exchange rates and annulization factors

Table 1: Larvicide Product Prices*

**Larvicide Product Prices**	Low	High	Mid-Point (at 2006 US$ prices)
Larvicide BTI VectoBac® WG (Kg)	20	30	25.84
Larvicide BTI VectoBac CG (Kg)	2.16	3.00	2.67
Larvicide BS VectoLex® WG (Kg)	50	70.00	62.02
Larvicide BS VectoLex® CG (Kg)	3.75	5.00	4.52

* Based on Fillinger U, Lindsay SW: Suppression of exposure to malaria vectors by an order of magnitude using microbial larvicides in rural Kenya. *Trop Med Int Health* 2006, 11:1629-1642.

Table 2: US Producer price index industry data “all other basic organic manufacturing sector” price inflator and deflators

Year	Prices	Inflator	Deflator
2003	100		
2004	106.4	1.064	0.93985
2005	121.5	1.1419173	0.87572
2006*	125.6	1.0337449	0.967357
2007**	128.8	1.0254777	0.975155

*base year for analysis
** data to end March 2007
Source: US Bureau of Labor Statistics http://data.bls.gov/PDQ/servlet/SurveyOutputServlet Accessed March 2007. Data no longer available online.

Table 3 Exchange Rates

**Exchange rate Average 2006**	**KES**	**US$1 buys**	**GB£1 buys**
**KES**	1.00	72.62	133.82
**US$**	0.01	1.00	1.84
**GB£**	0.01	0.54	1.00
	**TZS**	**US$1 buys**	**GB£1 buys**
**TZS**	1.00	1286.26	2373.57
**US$**	0.00	1.00	1.84
**GB£**	0.00	0.54	1.00

Table 4: Annualization Factors and Discount Rates

**Annualization factors**	**Useful life in years**				
Discount rate	2	3	5	8	15
3 percent	1.913	2.829	4.58	7.652	11.938

Annualization rate – Current value divided by annualization factor
