# Supplementary material for: Large-scale use of mosquito larval source management for malaria control in Africa: a cost analysis
Source: Malar J. 2011 Nov 8;10:338. doi: 10.1186/1475-2875-10-338 (PMC3233614; doi:10.1186/1475-2875-10-338)
Supplement: Additional file 2 — Dar es Salaam: Recurrent and capital unit costs. The file shows two tables itemizing the recurrent cost units and the capital cost units on which the economic costing is based. [file 1475-2875-10-338-S2.PDF]

Additional file 2:
Dar es Salaam: Recurrent and capital unit costs

Table 1: Dar es Salaam Recurrent Unit Costs

	Financial cost per unit		Allowances and extras		Units required	
**COST CATEGORY**	**TZS**	**US$**	**TZS**	**US$**	**Y0**	**Y1**
**INTERNATIONAL COSTS**						
**International Staff Time/Costs**						
External technical adviser		5,826		12,550	3	3
**NATIONAL COSTS**						
**Ministry of Health/NMCP Staff (Central & City Level)**						
**Staff Time/Costs**						
Central level NMCP/MoH staff -Director NMCP	1,250,000				0	0.025
Procurement Officer	700,000				0	0.019
City Medical Officer	1,250,000				0	0.019
**PROGRAM LEVEL COSTS**						
**Larviciding program Staff**						
**Staff Time/Costs**						
Program manager	13,000,000				1	1
City surveillance officers	7,200,000				2	2
Vector inspectors	1,813,333				0	6
Municipal coordinators	3,000,000				0	3
Ward supervisors	1,500,000				0	15
LCP	1,300,000				0	89
Larval surveillance CORPS	1,300,000				0	30
Drivers	5,500,000				3	3
Program administrator	2,000,000				1	1
Admin assistant	4,000,000				1	1
General hand	2,000,000				1	1
**Larvicide product, application equipment and transport**						
Larvicide BTI VectoBac™ WG (Kg)		25.84			0	0
Larvicide BTI VectoBac™ CG (Kg)		2.67			0	56,423.65
Weighing and packing of BS CG (bags and labor) per KG	1,796	0			0	5,442
Carriage Insurance Freight (CIF) on CG from U.S. to DSM (per container)		5,168		1,500	0	5
Port charges, storage and release fees		14,000			0	1
Import duty (25 percent of CIF) and VAT (20 percent of CIF + import duty)		52,529			0	1
Transport of Larvicides from Port to storage unit (inc dock hands)	1,000,000				0	1
Other field equipment (protective clothing, boots, buckets etc)	6,675,000				0	1
**Staff Training**						

	Financial cost per unit		Allowances and extras		Units required	
Salary, Transport & Lunch for LCP during training	7,500				0	810
Room hire for training	600				0	4
**Meetings and workshops/Community sensitization**						
Stakeholder meeting	5,000,000				0	1
Printing leaflets for distribution to households/communities	200				0	400000
**Operations costs and overheads**						
Office space rental City level	1,500,000				12	12
Utilities & Maintenance City Office (Electricity, Water)	75,000				12	12
Other overheads City office (e.g. Insurance and office furniture)	75,000				12	12
Storage and office space at municipal level	50,000				0	36
Utilities & Maintenance municipal offices (Electricity, Water)	2,500				0	36
Other overheads municipal offices (e.g. Insurance and office furniture)	2,500				0	36
Storage costs at ward level	30,000				0	180
Mobile phone credit for Manger and surveillance officer	30,000				48	48
Mobile phone credit for inspectors, coordinators and drivers	20,000				144	144
Mobile phone credit for ward supervisors	10,000				0	180
Telephone land line	200,000				12	12
Internet connectivity monthly flat rate	50,000				12	12
Stationary, printing, photocopying, office supplies	2,000,000				12	12
**Transport**						
Fuel insurance and servicing on vehicles	7,368,233				2	2
Motorbike fuel	3,257,226				0	9
Transport allowance for ward supervisors	260,000				0	15
**Adult Mosquito Monitoring**						
Human landing catches	5,000				3484	3484
Field equipment (cool boxes, cups, gauzes etc)	640,000				1	1
Lab tech salary	400,000				12	12

Table 2: Dar es Salaam Capital Unit Costs

	Purchase price		Number of Units	Useful life (years)
**CAPITAL COSTS (Useful life > 1 year)**	**TZS**	**US$**		
**Mapping area/breeding sites**				
Purchase of aerial photo	2,500,000		1	15
GIS consultant to map wards	1,900,000		1.5	15
Motorbike & operations costs for GIS consultant	4,857,226		1.5	15
GIS technician (assistant)	4,000,000		1.5	15
**Storage space & equipment**				
Purchase of container	1,000,000		1	8
Purchase of A/C unit	500,000		1	3
Lockable cupboards at ward level	50,000		15	8
Weighing scales at ward level	20,000		15	5
Water tank at ward level	20,000		15	8
**Vehicles**				
Project vehicle	15,000,000		2	5
Motorcycles	1,600,000		9	3
**Spray pumps**				
Hudson spray pumps		250	30	5
Granule blowers	330,000		6	5
**Computers, Mobile phones, GPS units and office equipment**				
Mobile phones		80	30	3
GPS units		120	6	3
Computer software and licenses		500	1	3
Desk Top computer		1,100	6	3
Laptop		1,300	1	3
Printer		500	1	2
**Adult Mosquito Monitoring Equipment**				
Microscope, light source		2,000	1	5
