# Supplementary material for: Large-scale use of mosquito larval source management for malaria control in Africa: a cost analysis
Source: Malar J. 2011 Nov 8;10:338. doi: 10.1186/1475-2875-10-338 (PMC3233614; doi:10.1186/1475-2875-10-338)
Supplement: Additional file 3 — Vihiga District: Recurrent and capital unit costs. The file shows two tables itemizing the recurrent cost units and the capital cost units on which the economic costing is based. [file 1475-2875-10-338-S3.PDF]

Additional file 3: Vihiga District: Recurrent and capital unit costs

Table 1: Vihiga District: Recurrent Unit Costs

	Financial cost per unit		Allowances & Extras	Units required			
				Scenario 1-WG		Scenario 2-CG	
**COST CATEGORY**	**TZS**	**US$**	**US$**	**Y0**	**Y1**	**Y0**	**Y1**
**INTERNATIONAL COSTS**							
**International Staff Time/Costs**							
External Technical Adviser		5,826	5,535	3	5	3	5
**NATIONAL COSTS**							
**Ministry of Health/NMCP Staff (Central, Provincial & District)**							
**Staff Time/Costs**	**Ministry of Health/NMCP Staff (Central, Provincial & District)**						
Director NMCP	790,440			0.019	1	0	0.019
NMCP Entomologist	308,736			0.019	1	0	0.019
Central level Procurement Officer	106,038			0.019	1	0	0.019
Provincial level Malaria Control Officer	206,796			0.019	1	0	0.019
District level Public Health Officer	206,796			0.025	1	0	0.025
District Medical Officer of Health	752,964			0.050	2	0	0.050
**PROGRAM LEVEL COSTS**							
**Larviciding program Staff**							
**Staff Time/Costs**	**Monthly wage (except LCP)**						
Program manager	25,728			6	12	6	12
Divisional Heads	17,233			36	72	36	72
Field (Location) Supervisor/s	8,837			0	156	0	156
LCP	250			0	19,800	0	19,800
Driver	9,873			6	12	6	12
Admin assistant	9,411			0	6	0	6
**Larvicide product, application equipment and transport**							
Larvicide BTI VectoBac™ WG (Kg)		25.84		0	0	0	7355.33
Larvicide BTI VectoBac™ CG (Kg)		2.67		0	0	0	183,883.33
Carriage Insurance Freight (CIF) on BTI WG from U.S. to Mombassa		11,744		0	1	0	0
Carriage Insurance Freight (CIF) on BTI CG from U.S. to Mombassa		70,782		0	0	0	1
Port clearance costs (agent and shipping line) per container		643.50		0	1	0	1
Taxes and tariffs - Exempted		0		0	1	0	1

Transport of Larvicides from Mombassa to Vihiga district HQ	185,575			0	2	0	13
Other field equipment (protective clothing, boots, buckets etc)	1,683,900			0	1	0	1
**Staff Training**							
Salary, Transport & Lunch for LCP during training	882,000			0	1	0	1
Training trip to DSM for Manager and Divisional heads	7,000			0	7	0	7
**Meetings and workshops/Community sensitization**							
Stakeholder meeting	5000			0	1	0	1
**Operations costs and overheads**							
Office space rental	6,000			6	12	6	12
Storage space rental district level (containers)	120,000			0	1	0	4
Utilities & Maintenance District Office (Electricity, Water)	1,500			6	12	6	12
Other overheads district office (e.g. Insurance and office furniture)	1,500			6	12	6	12
Storage and office space at divisional level	2,000			0	30	0	30
Utilities & Maintenance divisional offices (Electricity, Water)	500			0	5	0	5
Other overheads divisional offices (e.g. Insurance and office furniture)	125			0	5	0	5
Truck & driver daily rental to move product from district to division	10,000			0	4	0	8
Transport of product to field (LCP transport allowance)	250			0	19,800	0	19800
Mobile phone credits Manager/Div Head/Driver per month (8 people)	1,200			42	96	42	96
Mobile phone credits for Field supervisors + admin (21)	500			0	84	0	84
Internet connectivity monthly flat rate	3,000			6	12	6	12
Stationary, printing and photocopying	3,631			6	12	6	12
**Transport**							

Vehicle Fuel costs (KM) 1liters of diesel/10km, 1L = 63KES	6.3			13,000	26,000	13000	26000
Insurance	162,000			0.5	1	0.5	1
Vehicle Service	12000			2.60	5	2.60	5.20
Tires	60000			0.325	1	0.325	0.65
Shock absorbers & other repairs	15000			0.5	1	0.5	1
Motorbike Fuel costs (KM) 1L= 76KES; 1L = 40KM	1.90			78,000	156,000	78000	156000
Motorbike Insurance	6,000			3	6	3	6
Motorbike Maintenance/bike/month	2,000			36	72	36	72
Bicycles	4,000			0	26	0	26
**Adult Mosquito Monitoring**							
Traps (pots)	80			351	702	351	702
Supplies (ethanol, vials, Petri dishes, dissecting kits)	5,000			6	12	6	12

Table 2: Vihiga District: Capital Unit Costs

	**Purchase price**		**Units required**		**Useful life (years)**
**CAPITAL COSTS (Useful life > 1 year)**	**TZS**	**US$**	**Scenario 1 WG**	**Scenario 2 CG**	
**Vehicles**					
Project vehicle (Toyota Hilux Diesel pickup)	2,700,000		1	1	5
Motorcycles	100,000		6	6	3
**Spray pumps**					
Hudson spray pumps	18,155		400	0	5
**Computers, Mobile phones, GPS units and office equipment**					
Mobile phones		80	29	29	3
GPS units		120	6	6	3
Computer software and licenses		500	1	1	3
Desk Top computer		1,100	1	1	3
Internet connectivity		300	1	1	3
Laptop		1,300	1	1	3
Printer		500	1	1	2
**Adult Mosquito Monitoring**					
Microscope, light source		2,000	1	1	5
