# Supplementary material for: Large-scale use of mosquito larval source management for malaria control in Africa: a cost analysis
Source: Malar J. 2011 Nov 8;10:338. doi: 10.1186/1475-2875-10-338 (PMC3233614; doi:10.1186/1475-2875-10-338)
Supplement: Additional file 4 — Mbita Division: Recurrent and capital unit costs. The file shows two tables itemizing the recurrent cost units and the capital cost units on which the economic costing is based. [file 1475-2875-10-338-S4.PDF]

Additional file 4:
Mbita Division: Recurrent and capital unit costs

Table 1: Mbita Recurrent Unit Costs

	Financial cost per unit		Allowances and extras	Scenario 1-WG		Scenario 2-CG	
				Units required		Units required	
**COST CATEGORY**	**TZS**	**US$**	**US$**	**Y0**	**Y0**	**Y0**	**Y1**
**INTERNATIONAL COSTS**							
**International Staff Time/Costs**							
External Technical Adviser (UF)		5,826	3,690	1	2	1	2
**NATIONAL COSTS**							
**Ministry of Health/NMCP Staff (Provincial & District)**							
**Staff Time/Costs**	**Annual salaries**						
Provincial level Malaria Control Officer	206,796			0	1	0	1
District level Public Health Officer	206,796			0	1	0	1
District Medical Officer of Health	752,964			0	1	0	1
**PROGRAM LEVEL COSTS**							
**Larviciding program Staff**							
**Staff Time/Costs**	**Monthly wage (except LCP)**						
Program manager	25,728			12	12	12	12
Field Supervisor/s	17,233			36	36	36	36
Support staff (Drivers)	9,873			12	12	12	12
LCP	250			0	7280	0	7280
**NON-SALARY RECURRENT COSTS (by Activity/program area)**							
**Larviciding (intervention)**							
Larvicide BTI VectoBac™ WG (Kg)		25.84		0	582.4	0	0
Larvicide BTI VectoBac™ CG (Kg)		2.67		0	0	0	14560
CIF on BTI WG from U.S. to Mombasa (insurance below)		5,168.32		0	0.158347	0	0
CIF on BTI CG from U.S. to Mombasa (insurance below)		5,168.32		0	0	0	1.029345
Port costs (agent and shipping line) per container				0	1	0	1
Taxes and tariffs - Exempted				0	1	0	2
Transport of Larvicides from Mombasa to Mbita district	185,575			0	1	0	2
Insurance on Freight		1,500		0	1	0	2
Other field equipment (protective clothing, boots, buckets etc)	147,650			0	1	0	1

**Staff Training**							
Field staff training costs (food, per diems)	350			0	210	0	210
Staff training costs	7,000			0	4	0	4
**Meetings and workshops/Community sensitization**							
Stakeholder meetings	10,500			0	1	0	1
**Operating costs and overheads**							
Office space rental (monthly)	18,155			12	12	12	12
Storage space rental district level (container annual cost)	120,000			0	1	0	1
Mobile phone credits Manager/ supervisors/Driver per month (5 people)	1,200			60	60	60	60
Internet connectivity monthly flat rate	3,000			12	12	12	12
Stationary, printing and photocopying	3,631			12	12	12	12
**Transport**							
Vehicle Fuel costs (KM) 7.3KES per KM	7.3			13000	13000	13000	13000
Insurance @ 6 percent of vehicle value	162,000			1	1	1	1
Vehicle Service	12,000			2.6	2.6	2.6	2.6
Tires	60,000			0.325	0.325	0.325	0.325
Shock absorbers & other repairs	15,000			1	1	1	1
Motorbike Fuel costs (KM) 1L= 90KES; 1L = 40KM	2.25			39000	39000	39000	39000
Motorbike Insurance	6,000			6	6	6	6
Motorbike Maintenance/bike/month	2,000			3	3	3	3
Bicycles	4,000			0	28	0	28
**Adult Mosquito Monitoring**							
Traps (pots)	80			152	152	152	152
Supplies (ethanol, vials, Petri dishes, dissecting kits)	1,500			12	12	12	12

Table 2: Mbita Division: Capital Unit Costs

	Purchase price		Number of Units		
**CAPITAL COSTS (Useful life > 1 year)**	**KES**	**US$**	**Scenario 1 WG**	**Scenario 2 CG**	**Useful life (years)**
**Transport**					
Project vehicle (Toyota Hilux Diesel pickup)	2,700,000		1	1	5
Motorcycles	100,000		3	3	3
**Spray pumps**					
Hudson spray pumps	18,155		30	0	5
**Computers and other equipment**					
Mobile phones		80	5	5	3
GPS units		120	3	3	3
Computer software and licenses		500	1	1	3
Desk Top computer		1,100	1	1	3
Internet connectivity		300	1	1	3
Printer		500	1	1	2
Lockable cupboards for storage at field level	2,000		3	3	8
**Adult Mosquito Monitoring Equipment**					
Microscope, light source		2,000	1	1	5
